# Gene Networks and Metacommunities: Dispersal Differences Can Override Adaptive Advantage

**DOI:** 10.1371/journal.pone.0021541

**Published:** 2011-06-28

**Authors:** Jacob W. Malcom

**Affiliations:** Department of Integrative Biology, University of Texas at Austin, Austin, Texas, United States of America; Argonne National Laboratory, United States of America

## Abstract

Dispersal is an important mechanism contributing to both ecological and evolutionary dynamics. In metapopulation and metacommunity ecology, dispersal enables new patches to be colonized; in evolution, dispersal counter-acts local selection, leading to regional homogenization. Here, I consider a three-patch metacommunity in which two species, each with a limiting quantitative trait underlain by gene networks of 16 to 256 genes, compete with one another and disperse among patches. Incorporating dispersal among heterogeneous patches introduces a tradeoff not observed in single-patch simulations: if the difference between gene network size of the two species is greater than the difference in dispersal ability (e.g., if the ratio of network sizes is larger than the ratio of dispersal abilities), then genetic architecture drives community outcome. However, if the difference in dispersal abilities is greater than gene network differences, then any adaptive advantages afforded by genetic architecture are over-ridden by dispersal. Thus, in addition to the selective pressures imposed by competition that shape the genetic architecture of quantitative traits, dispersal among patches creates an escape that may further alter the effects of different genetic architectures. These results provide a theoretical expectation for what we may observe as the field of ecological genomics develops.

## Introduction

Bridging levels of organization is a key goal of biological research. We would like to understand the genotype-phenotype map (GPM) and the phenotype-environment map (PEM), with an ultimate goal of integrating across both mappings to comprehend the genotype-environment map (GEM). Progress towards this integration requires accounting for known dynamics at both sub-mappings. For example, it is thought at this time that the GPM is best represented as an interacting network of genes, proteins, and various environmental inputs, and this representation deviates from the standard, purely additive model of the GPM. Elucidating the GEM will require the derivation of a set of expectations for what should be observed at different levels of organization.

Ecologists and evolutionary biologists increasingly recognize that phenotypic evolution may alter ecological dynamics (and vice-versa). For example, Hairston and colleagues [1] developed a formal framework for partitioning variance between evolutionary and ecological causes, and provided worked examples in which evolution played a dominant or minor role. Fukami and colleagues [2] used *Pseudomonas fluorescens* to test how evolution shapes community assembly. Yoshida and colleagues [3], [4] and terHorst and colleagues [5] showed that prey evolution damped the effects of predators in rotifer-algae and pitcher-plant communities, respectively. These studies make important strides in the “Emerging synthesis between community ecology and evolutionary biology” [6], but are restricted to single, panmictic populations.

In contrast, many species exist in sets of patches connected by dispersal, a process that is well-known to affect both ecological and evolutionary dynamics. In an ecological setting, the fields of metapopulation ecology [7], [8] and metacommunity ecology [9]–[11] are dedicated to the interplay between population dynamics and dispersal (i.e., local and regional processes). Patches may be colonized by dispersing individuals (or propagules), while populations at other patches go extinct. Dispersal also provides the introduction of novel predators, prey, competitors, or other ecosystem engineers to local patches [12]–[17]. In an evolutionary setting, dispersal has similarly multifarious [18] effects. For example, dispersal may alternately drive swamping in which genotypes maladapted to a local environment are consistently introduced (i.e., migration load), breaking down locally-adapted genotypes, or dispersal may introduce adaptive alleles that increase fitness in a population [19]–[23].

Numerous researchers have examined the role of evolution in metacommunities, where the effects of dispersal on ecological and evolutionary dynamics should be important (see [24] for a review). As a recent empirical example, Venail and colleagues [25] used *P. fluorescens* in a metacommunity setting to show that even minimal dispersal increased both diversity and productivity as populations adapted to the different carbon sources. Three papers since the review are most-similar to the research described herein. Loeuille and Leibold used a three-patch model in which three species disperse between patches and compete for a common resource [26]. They found a strong interaction between rates of dispersal, mutation, and environmental change such that local adaptation could preclude invasion of a second species given low dispersal; local adaptation may not lead to exclusion at high dispersal rates; and community outcomes were contingent on the rates of environmental change and mutation. Similarly, Urban and De Meester used a three-patch model to further explore the effects of colonization order and adaptive speed on the outcome of a two-species community [27]. In their scenario, early colonization of a species with high adaptive speed will regularly lead to the exclusion of a subsequent colonizer, but this priority effect may be lost if the original colonizer possesses a lower adaptive speed than a subsequent colonizer. Urban and colleagues approached the problem of evolutionary ecology in metacommunities and, building on the work of Gomulkiewicz and Holt [28], found that trait heritability may be a major factor in driving the successful colonization of patches [11]. If there is a link between the genetic architecture of quantitative traits and trait heritability, then there should be a consistent mapping from the GPM to the PEM.

Study of the GPM is the purview of genetics, genomics, and related fields; the ecological implications of the research are generally not considered. Research from these fields are important, however, insofar as conceptualizing the GPM not as a purely linear, additive system, but rather, as complex networks of interacting genes, proteins, and environmental inputs [29], [30] with extensive epistasis [31], [32]. A few papers have considered the implications of different network structures on ecological dynamics. Kimbrell [33] and Kimbrell and Holt [34] examined the effect of network connectivity on adaptation to, and colonization of, one or more sink habitats. They found that lower connectivity generally results in faster adaptation. Repsilber and colleagues modeled gene networks of 3–10 genes and found smaller, less-connected networks resulted in faster adaptation and longer population persistence times for a single species in a heterogeneous landscape [35]. Malcom modeled gene networks from 16–256 genes in size and different topologies in a variety of ecological scenarios. He found that smaller, scale-free networks confer higher trait heritability than larger, random-topology networks, thus creating the link from genetic architecture to rates of phenotypic change (i.e., heritability). This cascades to drive population recovery after a sudden environmental change as well as population persistence in a constantly-changing environment [36], [37]. Another interesting finding is that there is an adaptation speed-accuracy tradeoff when two species compete [38]. Smaller networks permit faster adaptation, but larger networks permit high accuracy (and precision), which is an important aspect of trait evolution [39].

Here I consider a two-species, three-patch metacommunity similar to the scenarios examined by Loeuille and Leibold [26] and Urban and De Meester [27]. Different from their simulations, here, the GPM of each species is modeled as a gene network of 16, 64, or 256 genes arranged in a random or scale-free topology. The two species compete for a limiting resource with two characteristics, the first a quantity that limits the total number of individuals in a patch but whose replenishment rate is fast enough to prevent over-utilization. Second, the resource is characterized by a quality (e.g., palatability) that evolves through time; the gene network-encoded trait maps to this quality and must adapt in order for a population to persist. I first examine how different genetic architectures of the two species affect community outcomes in terms of persistence times and the probability of the focal species going to regional dominance. The major result is a distinct conflict between the roles of the gene network characteristics and dispersal rates: the probability of coexistence declines as the competing species diverges along either axis. I also compare how a single-species, multi-patch system (i.e., metapopulation) differs from the two-species, multi-patch system (i.e., metacommunity). I close with a discussion of the implications of these results for both genomics (and related fields) and evolutionary ecological research.

## Results

**Figure 1:**
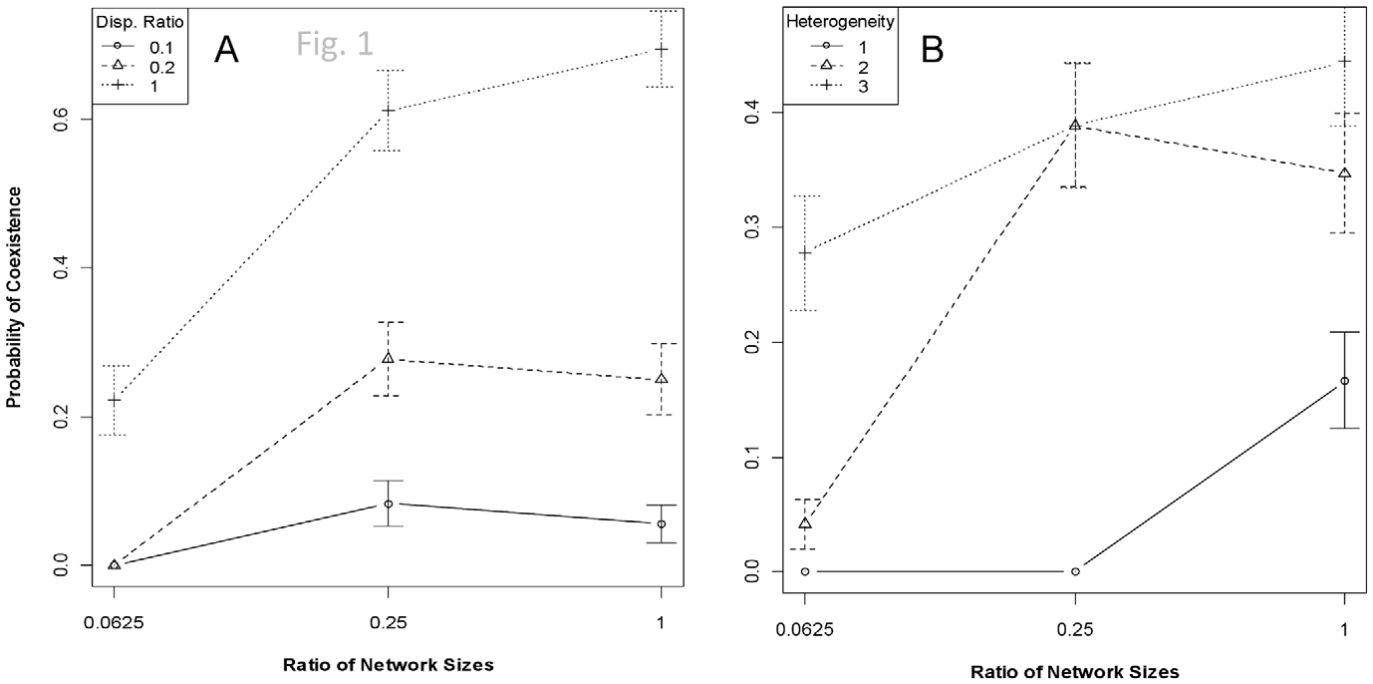
The probability of 2-species coexistence (±95% CI) in a metacommunity as a function of genetic architecture, dispersal ability, and landscape heterogeneity. Coexistence is defined as both species existing in the regional community after 750 generations of competition and dispersal among patches. Note the different y-axis scales between panels. Panel A. Coexistence is most-probable when neither species has either an adaptive advantage (i.e., genetic architectures are identical) or a dispersal advantage (i.e., dispersal rates are identical). Controlling for network size—i.e., looking vertically along the right-hand side of the figure—the prominent role of dispersal is readily apparent. Controlling for dispersal rates—i.e., following the dashed line with cross points—the role of different network sizes is readily apparent. Conversely, species reliably sort in the metacommunity when there is disparity in both dispersal ability and genetic architecture. Panel B. The probability of coexistence as a function of landscape heterogeneity and genetic architecture. Coexistence is most-likely when heterogeneity is highest (species can ‘find’ the patch for which they are best evolutionarily matched). When all patches are identical (heterogeneity1), long-term coexistence is most likely, but still relatively rare, when both species have identical genetic architectures.

**Figure 2:**
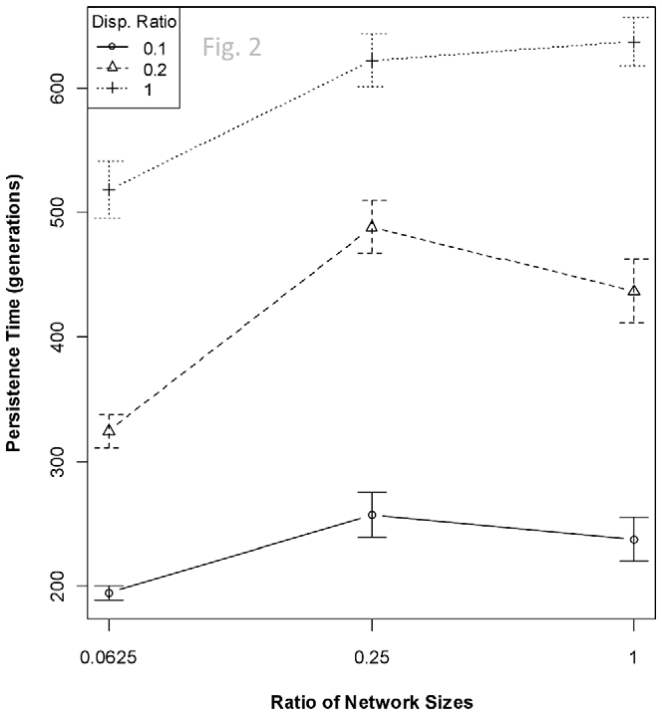
The duration of 2-species coexistence (±95% CI) in a metacommunity as a function of genetic architecture and dispersal ability. The duration of coexistence increases as dispersal rates and genetic architectures of competing species converge. The effect of differences in dispersal rate is generally greater than the effect of different genetic architectures. Local adaptive accuracy is higher in the species that disperses at a lower rate than the competitor, whereas the higher-dispersing species tends to adopt an “average” phenotype that does not confer an advantage.

In addition to the trade-off between speed and accuracy of adaptation as a function of genetic architecture–as is apparent in a single-patch scenario [38]—dispersal rates emerge as an important player in community dynamics when competition occurs among three disjoint patches. The probability that two species will coexist over the 750 generations of the simulations is largely a function of the relative dispersal ability of the species and the relative size of the underlying gene regulatory networks (Table 1). These relationships are conditional on the heterogeneity of the patches, i.e., whether the resource evolves at a single rate across all patches or up to three unique rates. When each species has the same size network, the probability of coexistence steadily increases as dispersal rates become equitable (Figure 1A, *right-hand side of figure*). Alternatively, when dispersal rates are identical between the two competing species, coexistence becomes more likely as the sizes of the networks converge (Figure 1A, *dashed line and crosses*). That is, as the species become more similar in either the genetic architecture of the limiting trait or their dispersal ability, neither is able to garner an advantage of adaptive speed, adaptive accuracy, or local adaptation over their competitor. The conditioning on heterogeneity is readily apparent in Figure 1B. The persistence of both species in a metacommunity is maximized when dispersal and network characteristics are most similar (Figure 2). Dispersal and network size explained 49% of the variance in persistence times (*P*<2.2e^−16^). Dispersal rate is the primary driver of persistence, followed in equal weight by the size of the network and the mutation rate (Table 2).

**Table 1:** Factors affecting the probability of coexistence, over 750 generations, in the metacommunity scenario.

Factor	Direction	df	Deviance	P-value
Competitor *d*	(-)	1	68.9	1.40e^−12^
Competitor *n*	(-)	1	34.5	0.007
Heterogeneity	(+)	1	22.1	0.02
Comp. *n* x Hetero.	( )	1	2.7	0.11
Residual		319	231.8	
Total		323	359.9	

*Direction* refers to the effect on coexistence: (-)inverse, (+)direct; *Heterogeneity* refers to the number of unique patch rates of environmental change (1, 2, or 3 unique); *d* is the dispersal rate; *n* is the number of genes in the network.

**Table 2:** Factors affecting the duration of coexistence in the metacommunity scenario.

Factor	Direction	% Variance	*P-*value
		Explained	
Competitor *d*	(-)	45	<2.2e^−16^
Competitor *n*	(-)	3	1.7e^−05^
Competitor µ	(+)	3	2.7e^−05^

*Direction* refers to the effect on coexistence: (-)inverse, (+)direct; *d* is the dispersal rate; *n* is the number of genes in the network; µ is the mutation rate.

**Figure 3:**
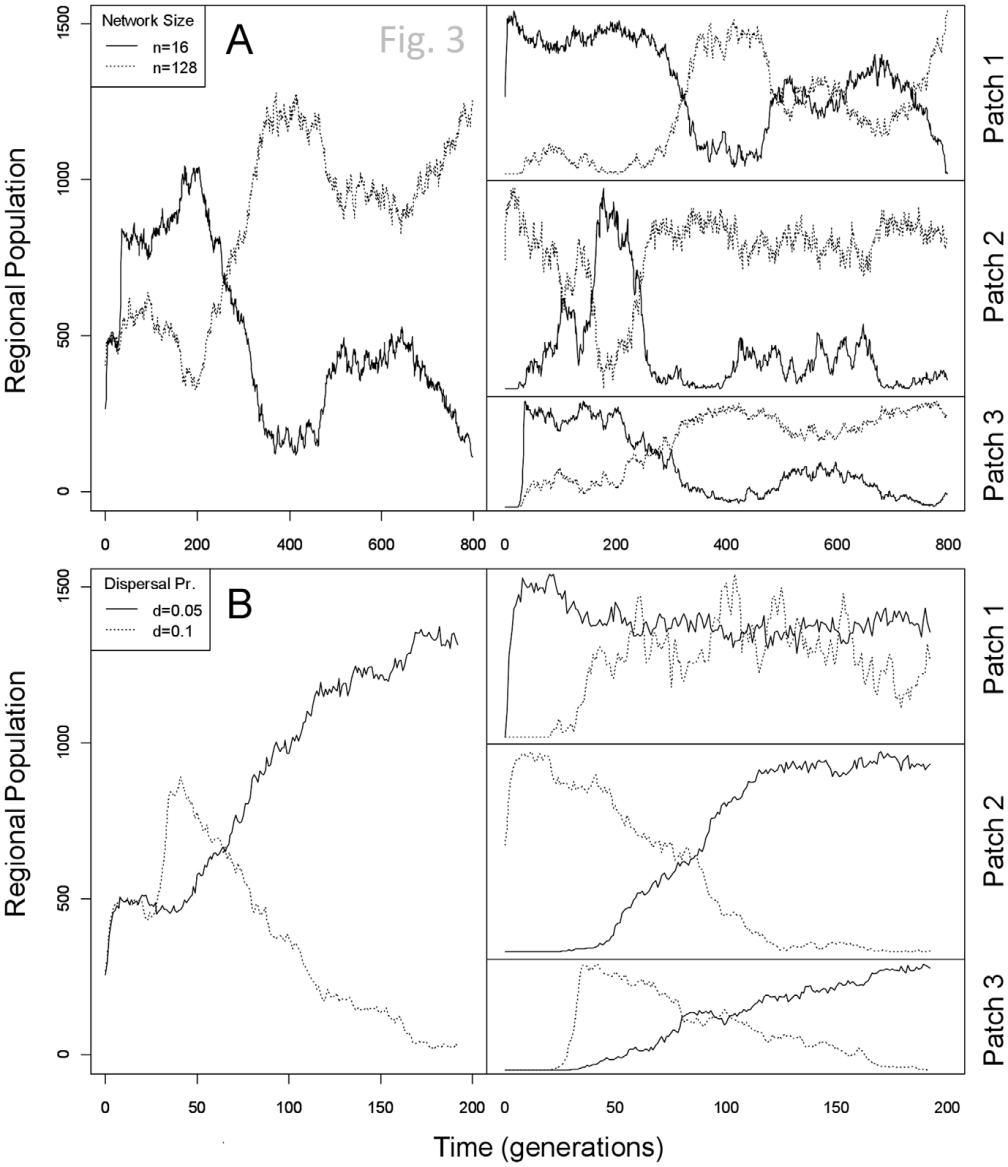
Two examples of runs in which priority effects are lost. Panel A shows that even though the faster-evolving species (network size16) arrives in Patch 3 before the slower-evolving species (network size128)—and for a period of time its regional population is considerably larger than the competitor—the greater adaptive accuracy of the slow-evolving species results in its regional dominance for the majority of the run. Panel B shows a similar situation, except that both species have the same genetic architecture (network size16, topologyscale-free, mutation rate1e^−3^, recombination rate0.05) but different dispersal probabilities. The high-dispersing species arrives at Patch 3 before the low-dispersing species and goes to local dominance, but the cost of high dispersal on adaptive ability eventually cancels the priority effect and the low-dispersing species outcompetes the high-dispersing species.

Priority effects, which have been observed in previous metacommunity simulations [27], could be lost in these simulations. In Figure 3A, both species possess the same dispersal probability, but the species with a small network arrives at Patch 3 before the second species (which possesses a larger, and more slowly-adapting, network). The first species' population increases rapidly, but the second species' population eventually surpasses the first species because it can adapt more accurately. Both species in Figure 3B possess the same size gene network (n16), but the species with the higher dispersal ability arrives in Patch 3 before the species with lower dispersal. The later-arriving species again goes to local dominance, presumably because its lower dispersal rate permits greater local adaptation. In a second set of simulations, I measured the environment-phenotype difference across all species and patches to quantify the effect of dispersal on adaptation. Higher dispersal rate reduced the mismatch between the trait and the evolving environmental variable (*p*<2.2e^−16^; Figure S1).

**Figure 4:**
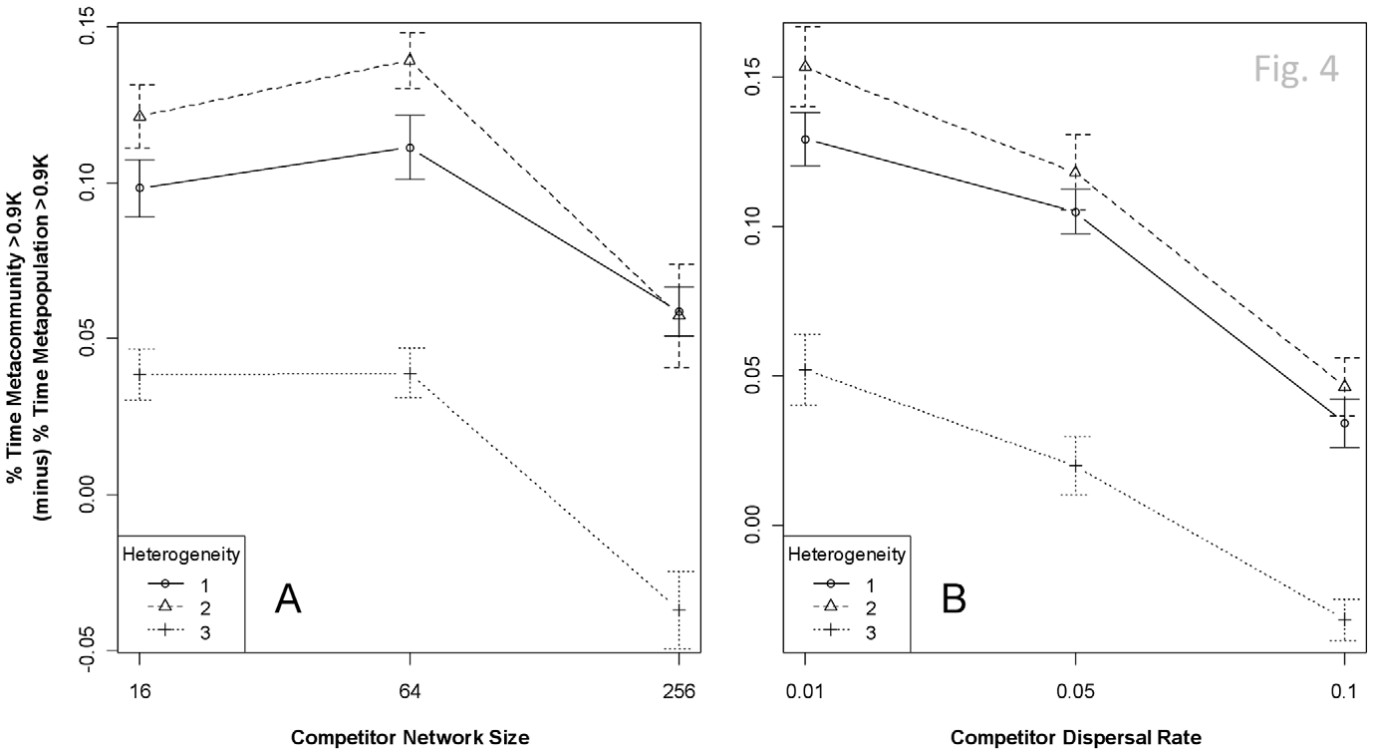
The effect (±95% CI) of a second species on the time that the regional population remains at or above 90% of carrying capacity. These figures represent a measure of ecosystem function to the extent that when all three patches are at capacity, the material and energy flows through the system should be maximized. The y-axis represents the difference in the percentage of time the regional population of a 2-species meta*community* spends >90% carrying capacity (>0.9 K) compared to the percentage of time that a single-species meta*population* spend >0.9 K. The presence of a second species with different genetic architecture tends to increase the amount of time that the regional population is >0.9 K, except when heterogeneity is high and the competitor has a very large network (Panel A). The advantage of increased regional population conferred by the presence of the competitor declines with the competitor's increasing dispersal probability, likely a reflection of the cost of dispersal on local adaptation (Panel B).

To compare the effects of the inclusion of a second species in the three-patch system, I consider the amount of time that the community spends above 90% of carrying capacity (0.9 K) as a proxy of ecosystems processes: the more time that populations are near capacity, the greater the energy or material flow through the system. The addition of a second species to a multi-patch system changes both the time required to reach 0.9 K and the time the community's population is greater than 0.9 K. First, as expected, time-to-0.9 K declines by 2.4 to 5.3 generations (*p*<0.0001) when comparing low-to-medium or low-to-high dispersal rates. Neither genetic architecture nor landscape heterogeneity has an effect on time-to-0.9 K (*p*0.12 and *p*0.6, respectively; whole model R^2^0.24). Second, network size, dispersal ability, and landscape heterogeneity all affect the proportion of time the community population size remained above 0.9 K (*p*<3.4e^−8^ for each term, model R^2^0.32). Two-species communities tend maintain larger populations for a longer period of time (mean difference0.088), but the difference is not statistically significant (SD0.11). The non-significance is driven primarily by the fact that the presence of a species with a large network (*n*256 genes) depresses the regional population in relation to mid- and small-sized networks across all landscape heterogeneities (3–11%, *p*<0.00014; Figure 4A). In contrast to the time-to-0.9 K, time-above-0.9 K declines steadily with increasing competitor dispersal rates (*p*1.7e^−12^, Figure 4B). Increasing landscape heterogeneity decreases the time-above-0.9 K when two species are present in the community, especially contrasting a homogenous landscape to either the two- or three-patch speed scenarios (*p*1.6e^−8^, Figure 4).

## Discussion

The interplay between genetic architecture, phenotypes, and evolutionary and ecological dynamics are complex, yet despite the rapid acceleration of biological research, a fundamental understanding of the interplay among these factors remains elusive. Progress is being made in refining the both the GPM and PEM, and as progress is made in empirically spanning these levels of organization, we need sets of theoretical expectations to unite the constituent pieces. Here I have attempted a step in that direction with a set of simulations that span from the gene regulatory network underlying a quantitative trait to an evolving resource quality to which the quantitative trait maps, with the added realism of competition and dispersal. Smaller and scale-free networks (in conjunction with lower recombination rates) tend to increase trait heritability [35], [36], which permits faster population recovery and longer population persistence times. Including a competitor in a single patch scenario, a tradeoff between adaptive speed and adaptive accuracy, driven by genetic architecture, is apparent [38]. By incorporating competition and dispersal in the current models, I have increased the degree of realism and refined expectations of what we should observe when linking genotypes to ecological and evolutionary dynamics.

I found that the strong opposing effects of genetic architecture and dispersal were readily evident, similar to the results obtained by Loeuille and Leibold [26]. I focused on network size, and the ‘rule-of-thumb’ that emerges is that if the ratio of species' dispersal rates is greater than the ratio of network sizes, then dispersal drives the community outcome. Conversely, if the ratio of network sizes is greater than the ratio of dispersal rates, then genetic architecture drives community outcome. This ‘rule’ is conditional on the rates of change of the resource quality, which interacts strongly with network architecture as discussed previously [37], [38]. As expected, high-dispersing species tend to colonize the new patch quickly, but gene flow impedes local adaptation. Thus, when genetic architectures are similar, and even when the low-dispersing species disperses only at 1% per generation, the rate is sufficient to allow the colonization of the third patch and out-adapt the initial colonizer who suffers the swamping effects of high dispersal. Conversely, differences in genetic architecture can over-ride differences in dispersal ability, and the ‘best’ match between genetic architecture and rate of environmental change is found. These results suggest that the evolvability of limiting traits may over-ride dispersal effects and vice-versa.

Theories of community assembly and dynamics fall along a spectrum of mechanisms from species sorting to neutral theory [40]–[43]. Malcom [38] proposed that network size for particular traits could be a novel axis of species sorting, such that there is sorting according to a species adaptive capacity, as determined by gene network characteristics, in addition to selection in the current environment. At the same time, evolution of network size towards a common, optimal size should be an equalizing mechanism that permits long-term persistence of competing species. As Leibold and Chase [42] and Loreau [44] have highlighted, dispersal is likewise a niche axis that can describe the relationship to a limiting resource such as space. These concepts are supported by the present results where network size and dispersal are distinct niche axes.

While priority effects similar to those discussed by Urban and De Meester [27] occurred in these simulations, the priority could be over-ridden by the competing effects of superior adaptive speed or accuracy. Urban and De Meester describe the role of faster adaptation in erasing priority effects, but the role of higher adaptive accuracy erasing an early arrival advantage, as in Figure 3A, has not been previously found to my knowledge. The result is not particularly surprising in light of the results of the single-patch competitive scenario where at sufficiently slow rates of environmental change the higher adaptive accuracy of large networks confers an advantage [38]. In addition to the tradeoff between adaptive speed and accuracy, these simulations showed the disadvantage that high dispersal can play, such as in the case of Figure 3B. Here, although the two species have the same adaptive potential as controlled by genetic architecture, the higher-dispersing species arrives first in patch 3 only to lose the timing advantage because the high migration reduces adaptive potential. This latter result coincides, on the evolutionary side, with well-established theory on the effects of dispersal [21] and is in agreement with the results of Urban and De Meester.

The role of biodiversity in maintaining ecosystem function has received attention since at least the 1970s, and interest has recently increased as we become aware of the scope of anthropogenic change [45]–[48]. In general, both theory and empirical research suggest a positive relationship between biodiversity and ecosystem function. Loreau has divided the mechanisms responsible for this pattern into (non-exclusive) selection and complementarity effects [44]. Although I did not model an ecosystem process here, we can generally assume that the greater the total number of individuals in a region, the greater the flux of materials and energy. The systematic increase in total number of individuals in the regional population in these simulations is an example of both complementarity and selection. In a single-species metapopulation, patches may remain uninhabited (or inhabited at a lower density) because of mismatches between adaptive capacity and rates of environmental change. The addition of a second species, however, tends to increase the time the population remains above 90% of the maximum observed population because the adaptive capacities of the species are complementary. The total regional population is highest when landscape heterogeneity matches the number of species (i.e., the resource quality changes at two rates). However, total regional population is systematically lower than two-rate scenarios (but typically higher than metapopulation scenarios) when the resource quality changes at a different rate in each patch.

The results suggest that even low dispersal is homogenizing the regional population slightly and reducing local adaptation. The homogenization hypothesis is reinforced by the point that total regional populations are depressed at higher dispersal rates (Figure 4B). Note that this result is found even though only one of the two species is dispersing at a higher rate. These outcomes are consistent with the empirical results of Venail and colleagues [25], who found that ecosystem function declined with increasing dispersal in *Pseudomonas* communities. Because I did not explicitly model nutrient flows, however, the source-sink effects of nutrient movement were not recovered as in analytical metaecosystem models [46], [49].

How do these results compare to the real world? The short answer is, we don't know (see [36], [38] for further elaboration). The technological advances required for resolving the GPM are too new for a knowledge base to have been established. A first order of business is estimating the gene networks underlying quantitative traits in various species to see if, in fact, there is considerable variation in the size or topological characteristics of the networks. An alternate hypothesis (and perfectly reasonable in the absence of empirical evidence) is that any particular challenge requires approximately the same size network regardless of the species in question. Ultimately, either result—i.e., very similar network sizes or different network sizes for a given trait—from data would be interesting and informative, even if the latter makes the results herein irrelevant. The next step down the line, to conduct experiments in multiple-patch metacommunities with species whose genetic architectures (for the relevant traits) are known, is a distant prospect. Even once it is possible, the inherent difficulties of metacommunity experiments will only be exacerbated.

In addition to our lack of data to confirm this work, we have to consider that these models, like all models, are simplifications of reality. The same caveats as highlighted by Malcom [36] apply: Boolean regulatory networks gloss over real differences of gene function, the details of which are interesting and may have important ramifications; the networks I use here are further simplified in that each gene is regulated by a single upstream factor, whereas real genes are often multiply regulated; there is ample evidence of widespread pleiotropy between real networks, and the traits that these linked networks underlie may be under different selection regimes. Lastly, the competition and dispersal scenarios considered here are greatly simplified, and other (non-network) research has shown the multi-species and multi-trophic scenarios can alter eco-evolutionary trajectories in unpredictable ways [50]. Including >2 species, and/or two or more trophic levels, with the GPM defined as complex networks could further refine our expectations of the links across the GEM. Lastly, I considered dispersal as a fixed trait, but the phenotypic features that enhance or dampen dispersal rates will often be evolvable [51], [52]. How does evolution of dispersal ability change the role of the genetic architecture of quantitative traits in ecological dynamics?

The primary conclusion to be drawn from these results is that both the genetic architecture of a trait critical to competition and dispersal rates of individuals can play important roles in determining metacommunity dynamics. If the ratio of network sizes (or some general measure of complexity that incorporates network topology) of two species is greater than the ratio of their dispersal rates, then the differences in adaptive capacity that the networks afford should drive community outcomes. Alternatively, if the ratio of dispersal rates is greater than the ratio of network size, then the system will be dispersal-driven, overriding the adaptive capacities of each species. Conversely, we should expect that ecological dynamics, such as those exhibited in metacommunities, will shape the genetic architecture of the traits that mediate dynamics. For example, selection should favor evolution towards a particular network size for long-term coexistence. These results provide a set of theoretical expectations that can be empirically tested.

## Materials and Methods

### Gene Network Model

I focus on individuals of two species competing in a three-patch metacommunity for a resource whose quality fluctuates through time at a variety of rates. Individuals of both species possess a single quantitative trait that maps to the quality of the limiting resource (see Introduction). The trait is encoded by a directed Boolean network of *n* genes (genetic architecture parameters in Table 3), with the on-off state of each determined dynamically. The topology of the competitor's network is initiated as either random (no preferential attachment) or scale-free (with preferential attachment) in its out-degree distribution [30]. Randomly-connected networks show an approximately Poisson degree distribution, whereas scale-free networks exhibit a power law degree distribution [53]. I use a lottery model algorithm to form the scale-free networks, i.e., the probability of an existing gene acquiring a connection to a new gene is proportional to the number of existing connections [53]. Network size and topology treatments, as well as other genetic architecture parameters, for both the focal and competitor species are given in Table 3.

**Table 3:** Basic experimental design of the metacommunity scenario.

	Network Size	Network Topology	Mutation Rate	Recombination
Focal species	16	scale-free	1e^-4^	0.05
Competitor	16, 64, 256	scale-free, random	1e^−3^, 1e^−4^, 1e^−5^	0.05, 0.5

**Figure 5:**
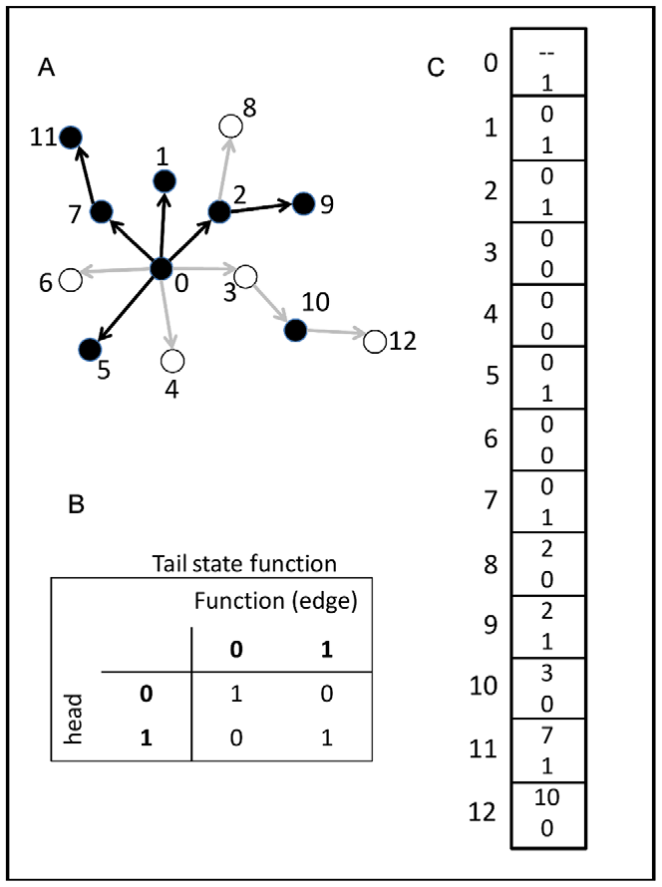
An example network, functional map, and chromosome. Panel A shows an example 13-gene Boolean network. Black nodes are up-regulated (“on”; state1) genes and white nodes are down-regulated (“off”; state0). If an edge connecting two nodes is black, the “head” gene (upstream) activates the “tail” gene (downstream), and if an edge is gray, the head represses the tail gene. Panel B provides the functional map; for example, if the head gene is “off” and the edge connecting the head and tail genes is an activator, then the tail gene is off (upper-right quadrant). Panel C shows the chromosome corresponding to the network in Panel A. Each block represents a gene (numbers along the left-hand side); within each block, the top number defines the “head” (i.e., immediately-upstream) gene while the bottom number defines the functional relationship (e.g., if 0, then the head gene is a repressor).

At the start of a run, every individual's network is randomly determined, as guided by the constraints of topological specification. With these relatively small populations, it is very unlikely that any two individuals possess the same exact network at simulation initiation. The binary state [0, 1] of each gene in the network except the upstream-most is determined by comparing the state of the gene immediately upstream to the functional relationship of the gene pair (Figure 5a, encoded by chromosome of 5c). The state of the upstream-most gene is determined randomly for each individual at simulation initiation, and is then inherited for subsequent generations. Some genes may act as repressors and others as activators, and the state of the downstream gene is determined by the match or mismatch between the state of the upstream gene and the function (Figure 5b). For example, if the upstream gene is “on” (state1) and is a repressor (function0), then the downstream gene takes the “off” state (state0). Alternatively, if the upstream gene state is 0 and it is a repressor, then the downstream gene takes the “on” state. Each gene except the basal-most has a single input to ease computational requirements (the number of calculations increases according to 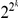 with *k* inputs [54]), but may have one or more outputs (i.e., may be pleiotropic). All network information is stored on a single chromosome consisting of two parts (Figure 5c). First, the topology is defined by a “tails list” of the downstream genes; the “heads list” (the controlling, upstream genes) is inferred from the index position of each tail list element. The relationship between heads and tails genes is randomly determined at the start of a simulation run, but, as noted above, the out-degree distribution is constrained by the scale-free versus random topological assignment. Figure 5a is an example 13-gene network whose states have been calculated given the information from the chromosome in Figure 5c.

Each individual's phenotype is determined by summing the states of all terminal genes in the network, i.e., genes with out-degree0, and scaling the value to the range of the environment (140). For example, the network in Figure 5a possesses eight terminal genes, four of which are “on”, thus the individual possesses a phenotype of 70 ( =  (140/8) * 4). I am thereby assuming that there are no biochemical limits given a particular network size: individuals with a 16-gene network can approximate a phenotype of 140, as can individuals with a 256-gene network. The consequence for this re-scaling is that smaller networks have lower resolution than larger networks, which is a reasonable assumption given that dividing any particular task among fewer actors will result in lower overall accuracy. I stored the phenotypes of each individual's parents and used mid-parent regression to estimate the trait's heritability in the population. Additive genetic variance was derived by multiplying the phenotypic variance by the heritability.

Each individual's phenotype is translated to a fitness relative to the environment using a Gaussian function of the form,
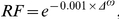



where Δ is the absolute value of the difference between the environment and the individual's phenotype, and ω is a value that changes the breadth of the selection function. That is, I assume that the environmental effect is absolute and the phenotypic variance of the population plays no role in how an individual is selected. I fixed ω at 2 in these simulations, based on the results of [36], [37]. Each individual's *RF* does not affect the number of offspring produced, but does affect the probability that an individual will survive to reproduce.

Individuals are sexually-reproducing hermaphrodites who mate at random. The number of offspring from a mating is determined by drawing a random value from a Poisson distribution with λ1.5. Gametes undergo recombination during a diploid meiotic stage to create an offspring chromosome that is a mixture of parental alleles, which in this model are the tails list and the functional relationships. The first element of the offspring chromosome is chosen from the first element of one parent, then subsequent elements are taken from the same parent until a random uniform number less than the recombination rate is drawn, at which point the element is drawn from the opposite parent. This continues the length of the chromosome. Mutation, as determined by testing a uniform random number against the mutation rate for each chromosomal element, occurs after the new chromosome is created. Although these mutation rates appear high, as noted by Frank [54], the effective mutation rate is about one order of magnitude lower because the trait is directly related to fitness. All mutations are non-synonymous and may affect either the controlling function of a gene (an activator mutates to suppressor) or the relationship to another gene (i.e., alter network topology).

Death occurs after reproduction in three stages. First, all parents are killed to prevent over-lapping generations. Next, the new generation is culled according to each individual's relative fitness: if the *RF* is less than a uniform random number, then the individual dies. This is the step in which competition is operationalized: if one species' mean fitness is higher than the second species', then fewer individuals of the first species will die and the species gains a numeric advantage. Last, a carrying-capacity is enforced by randomly killing individuals to bring the population below K200 in each of the three patches, for a regional number of individuals≤600.

### The Metacommunity

The basic experimental design of the metacommunity simulations follows the single-patch scenario [38] in that the focal species is held constant and the competitor takes different genetic architectures, with the added factor of differing dispersal abilities (Table 3). The resource quality variable is initialized at the same value (70 units) for all three patches, but each rate of resource quality change in each patch is 1e^−3^, 2.5e^−4^, or 1e^−4^ units per generation. Note that these rates are slightly different from those used in the single-patch competition scenario of Malcom [38], but cover a similar same range of values. Three metacommunity-level patch heterogeneities are possible: all patches change at the same rate, two change at the same rate, or all three change at different rates. Two patches are initialized each with 100 individuals of a single species. After 20 canalization generations in their starting patch, both species may begin dispersing at their given rate (Table 3). Review of additive variance plots from [38] showed that canalization tended to occur by the 15^th^ generation, and fluctuated around the stable mean through generation 20, hence the decision for 20-generation canalization. Note that the additive and phenotypic variances are not exactly equal, but results from [37] showed that the effect of the differences was very small compared to the effect of network size.

An individual disperses at a random angle and a random distance of 0–100 units if a random uniform number is less than the dispersal rate (0.1, 0.05, or 0.01). The three patches are equidistant and spaced at 50 units from edge to edge. Individuals die immediately if they do not land on one of the three patches. Here I am considering passive dispersers, such as seeds or zooplankton resting-stage eggs, rather than organisms that select patches to colonize. The simulations encompassed a full-factorial design of genetic architectures and rates of environmental change across the three patches. Each simulation continued 750 generations or until one of the two species went extinct.

Analyses were broken into two major groups. First, I examined the probability of both species coexisting in the metacommunity at 750 generations using a generalized linear model with a binomial distribution and logit link function. I then examined how persistence time was influenced by characteristics of the genetic architecture of the competing species and the rates of environmental change across patches using a linear model. Second, I compared a single-species scenario (i.e., metapopulation) to the two-species scenario (i.e., metacommunity). I focus on two metrics of the effects of a second species using the three patches: the time required for the community to reach 90% of the maximum observed regional population, and the percentage of time that the community remains above 90% of carrying capacity (0.9 K). The first metric is a joint measure of different dispersal rates and adaptability to the patches. The second metric may be considered a measure of ecosystem function, assuming that higher regional populations result in greater throughput of material or energy. I used the same model for the metapopulation as used in the metacommunity simulations, but kept only the focal species (with genetic architecture as in Table 3). I ran three replicate runs at each of the seven unique landscape combinations. I set the critical population size as 0.9 times the maximum regional population observed across all simulations, then extracted the first time (generation) at which the regional population was greater than the critical population size. Second, I extracted the percentage of time the regional population spent above the critical population size. I calculated the mean of time-to-0.9 K and proportion of time above 0.9 K for each of the seven unique combinations of rates of environmental change among the three patches for both the metapopulation and metacommunity scenarios. To draw contrasts for time-to-0.9 K and time-above-0.9 K, I subtracted the mean value from the metapopulation simulations from each run of the metacommunity simulations, matched for landscape arrangement. I then used a set of linear models to relate characteristics of the competitor species (genetic architecture, dispersal ability) to the change in time-to-0.9 K and time-above-0.9 K.

I used NetLogo 4.1[55] for all simulations; the model code is available in Text S1. All statistical analyses were conducted in R 2.10 [56]; I used Tukey's HSD [57] for post-hoc tests and Akaike's Information Criterion for model selection [58] where necessary.

## Supporting Information

Figure S1 **Mean difference between average phenotype and resource quality (i.e., the optimum) as a function of competitor dispersal rate.** Higher dispersal leads to regional homogenization such that, on average, the difference between trait value and the environment is greater. The mean differences were calculated as the absolute value of the average trait value in each patch minus the resource quality in each patch, and weighted according to the number of individuals (competitors) in the patch.
(TIF)

Text S1 **The NetLogo code for the metacommunity simulations.** Note that the species are named buggles and wuggles, rather than Sp1 and Sp2, just for fun.
(DOC)
