# Supplementary figures and images for: Gene Networks and Metacommunities: Dispersal Differences Can Override Adaptive Advantage
Source: PLoS One. 2011 Jun 28;6(6):e21541. doi: 10.1371/journal.pone.0021541 (PMC3125243; doi:10.1371/journal.pone.0021541)

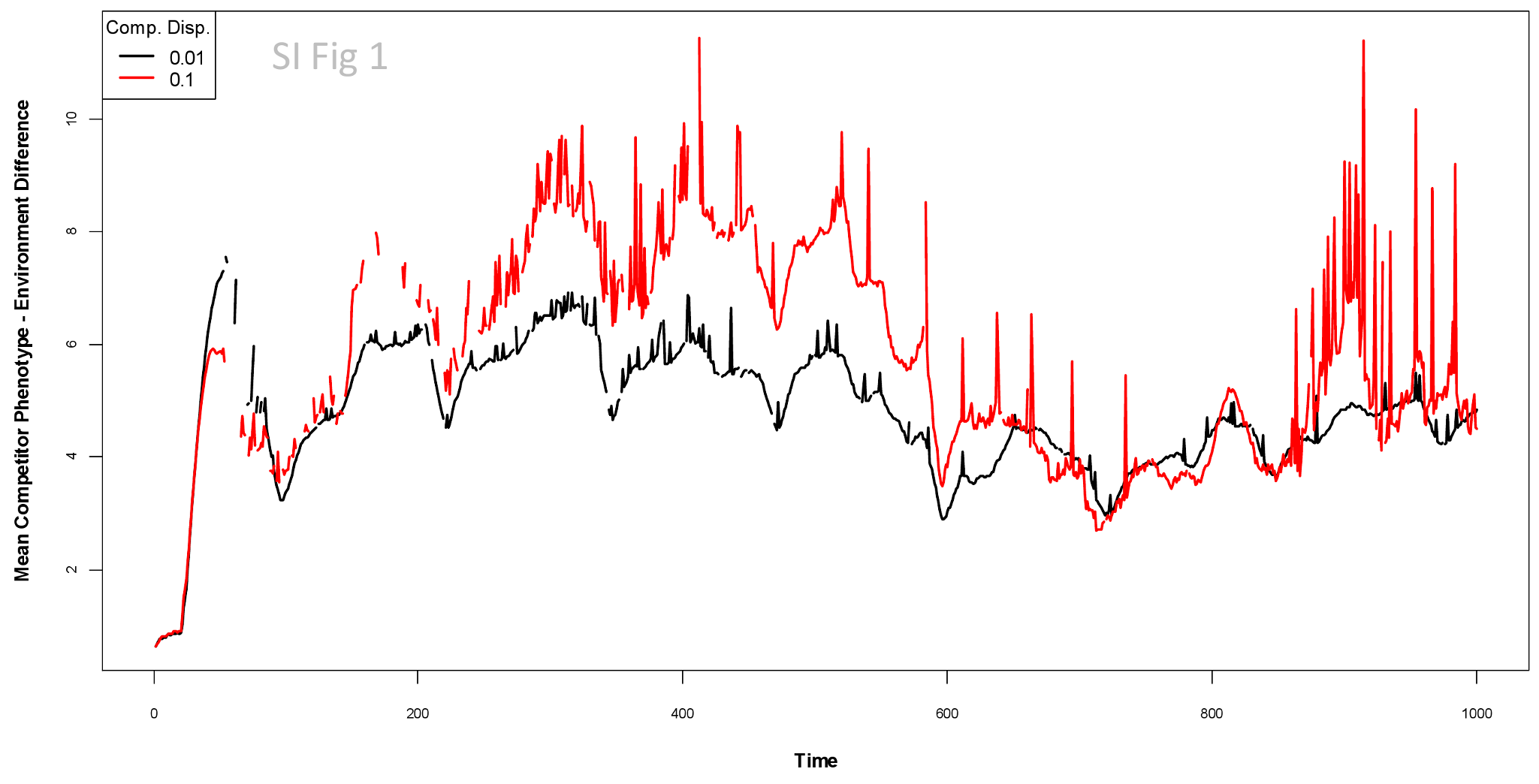

Supplement: Figure S1 — Mean difference between average phenotype and resource quality (i.e., the optimum) as a function of competitor dispersal rate. Higher dispersal leads to regional homogenization such that, on average, the difference between trait value and the environment is greater. The mean differences were calculated as the absolute value of the average trait value in each patch minus the resource quality in each patch, and weighted according to the number of individuals (competitors) in the patch. (TIF) [file pone.0021541.s001.tif]
