# Supplementary material for: Gene Networks and Metacommunities: Dispersal Differences Can Override Adaptive Advantage
Source: PLoS One. 2011 Jun 28;6(6):e21541. doi: 10.1371/journal.pone.0021541 (PMC3125243; doi:10.1371/journal.pone.0021541)
Supplement: Text S1 — The NetLogo code for the metacommunity simulations. Note that the species are named buggles and wuggles, rather than Sp1 and Sp2, just for fun. (DOC) [file pone.0021541.s002.doc]

breed [ buggles buggle ]

breed [ wuggles wuggle ]

globals [ Site1 Site2 Site3

S1-oneB S1-twoB

S1-oneW S1-twoW

S2-oneB S2-twoB

S2-oneW S2-twoW

S3-oneB S3-twoB

S3-oneW S3-twoW

S1-loc-B S1-imm-B

S1-loc-W S1-imm-W

S2-loc-B S2-imm-B

S2-loc-W S2-imm-W

S3-loc-B S3-imm-B

S3-loc-W S3-imm-W

Bphen-list

Bavg-phen

Bphen-var

Bunique

B_unP

Bmp-list

Bnumerator

Bdenominator

Wphen-list

Wavg-phen

Wphen-var

Wunique

W_unP

Wmp-list

Wnumerator

Wdenominator

BH2

Bavg-MP

WH2

Wavg-MP

BVp

WVp

BVg

WVg

BH2run

WH2run

BavgH2

WavgH2

bug_S1_P

bug_S2_P

bug_S3_P

bug_S1_Vp

bug_S2_Vp

bug_S3_Vp

wug_S1_P

wug_S2_P

wug_S3_P

wug_S1_Vp

wug_S2_Vp

wug_S3_Vp ]

patches-own [ pH

site-ID

increasing? ]

turtles-own [ age

homeland

network-size

mean-offspring

scale-free?

mutation-rate

recombination-rate

tails

heads

fx

G

chrom

genotype

d=1

od=0

od=max

phenotype

P

rel-fit

recomb

mother

father

midP

num1

den1

local ]

;;--------------------------------------------------------------------

to setup

ca

setup-landscape

setup-breeds

create-topo

set BH2run []

set WH2run []

end

to setup-landscape

ask patches

[ set pcolor green ]

set Site1 patches with [ (pycor > 20 and pycor < 40) and (pxcor > 20 and pxcor < 40) ]

ask Site1

[ set pcolor cyan

set pH Site1-pH

set site-ID 1

set increasing? true ]

set Site2 patches with [ (pycor > 20 and pycor < 40) and (pxcor > -40 and pxcor < -20) ]

ask Site2

[ set pcolor yellow

set pH Site2-pH

set site-ID 2

set increasing? true ]

set Site3 patches with [ (pycor > -50 and pycor < -30) and (pxcor > -10 and pxcor < 10) ]

ask Site3

[ set pcolor white

set pH Site3-pH

set site-ID 3

set increasing? true ]

ask patches with [ pcolor = green ]

[ set pH 1000 ]

end

to setup-breeds

ask Site1

[ sprout-buggles (random 3)

[ set color blue

set size 1

set network-size buggle-network-size

set mean-offspring buggle-mean-offspring

set recombination-rate buggle-recombination-rate

set mutation-rate buggle-mutation-rate

set homeland [ site-ID ] of patch-here ] ]

ask Site2

[ sprout-wuggles (random 3)

[ set color red

set size 1

set network-size wuggle-network-size

set mean-offspring wuggle-mean-offspring

set recombination-rate wuggle-recombination-rate

set mutation-rate wuggle-mutation-rate

set homeland [ site-ID ] of patch-here ] ]

end

to create-topo

ask turtles

[ set age ticks

set tails n-values network-size [ ? ]

set heads n-values 1 [ 0 ]

repeat (network-size - 1)

[ set heads fput "#" heads

set heads reverse heads

set heads replace-item (position "#" heads) heads (wookie)

set heads reverse heads ]

set heads reverse heads

set fx n-values network-size [ random 2 ]

set G random 2

set chrom (sentence heads fx G)

;;calculate genotypes

set genotype n-values 1 [ G ]

repeat (network-size - 1)

[ set genotype fput "#" genotype

set genotype reverse genotype

set genotype replace-item (position "#" genotype) genotype (read)

set genotype reverse genotype ]

set genotype reverse genotype

;;calculate phenotypes

set d=1 n-values network-size [ ? ]

foreach d=1 [ ifelse (member? (item ? tails) heads)

[ set d=1 replace-item ? d=1 0 ]

[ set d=1 replace-item ? d=1 1 ] ]

set phenotype (map [ ?1 * ?2 ] d=1 genotype )

set od=0 sum d=1

set P (round (sum phenotype * (140 / (network-size * 0.66) )))

;;calculate relative fitness

let delta (abs (P - [ pH ] of patch-here))

set rel-fit ( exp ((- 0.001) * (delta ^ 2)) ) ]

end

to-report wookie

let $t sublist tails 0 (position "#" heads)

let $t2 heads

let $t3 remove "#" $t2

let prefattach sentence $t $t3

report one-of prefattach

end

to-report read

let $h item (position "#" genotype) heads

let $f item (position "#" genotype) fx

let $s item $h genotype

ifelse (($s = 1 and $f = 1) or ($s = 0 and $f = 0))

[ report 1 ]

[ report 0 ]

end

;;-------------------------------------------------------------------

to go

if (count buggles <= 1) or (count wuggles <= 1) or (ticks = sim-length) [ stop ]

env-fluct

ask Site1 [ if S1-fluctuate [ S1-fluct ] ]

ask Site2 [ if S2-fluctuate [ S2-fluct ] ]

ask Site3 [ if S3-fluctuate [ S3-fluct ] ]

pair-up

GP

death

ask turtles [ move ]

if (ticks > 20) [ disperse ]

kill-program

summary-info

set BH2run fput BH2 BH2run

set WH2run fput WH2 WH2run

set BavgH2 mean BH2run

set WavgH2 mean WH2run

tick

end

to env-fluct

ifelse (ticks < 21)

[ set S1-fluctuate false

set S2-fluctuate false

set S3-fluctuate false ]

[ set S1-fluctuate true

set S2-fluctuate true

set S3-fluctuate true ]

end

to S1-fluct

ifelse (increasing? = true)

[ S1-increase-pH ]

[ S1-decrease-pH ]

end

to S2-fluct

ifelse (increasing? = true)

[ S2-increase-pH ]

[ S2-decrease-pH ]

end

to S3-fluct

ifelse (increasing? = true)

[ S3-increase-pH ]

[ S3-decrease-pH ]

end

to S1-increase-pH

ifelse (pH <= (140 - (140 * S1-speed)))

[ set pH (pH + (140 * S1-speed)) ]

[ set increasing? false ]

end

to S1-decrease-pH

ifelse (pH >= (0 + (140 * S1-speed)))

[ set pH (pH - (140 * S1-speed)) ]

[ set increasing? true ]

end

to S2-increase-pH

ifelse (pH <= (140 - (140 * S2-speed)))

[ set pH (pH + (140 * S2-speed)) ]

[ set increasing? false ]

end

to S2-decrease-pH

ifelse (pH >= (0 + (140 * S2-speed)))

[ set pH (pH - (140 * S2-speed)) ]

[ set increasing? true ]

end

to S3-increase-pH

ifelse (pH <= (140 - (140 * S3-speed)))

[ set pH (pH + (140 * S3-speed)) ]

[ set increasing? false ]

end

to S3-decrease-pH

ifelse (pH >= (0 + (140 * S3-speed)))

[ set pH (pH - (140 * S3-speed)) ]

[ set increasing? true ]

end

to pair-up

if any? turtles-on Site1

[ set S1-oneB sort [ who ] of buggles-on Site1

set S1-twoB shuffle S1-oneB

set S1-oneW sort [ who ] of wuggles-on Site1

set S1-twoW shuffle S1-oneW ]

if any? turtles-on Site2

[ set S2-oneB sort [ who ] of buggles-on Site2

set S2-twoB shuffle S2-oneB

set S2-oneW sort [ who ] of wuggles-on Site2

set S2-twoW shuffle S2-oneW ]

if any? turtles-on Site3

[ set S3-oneB sort [ who ] of buggles-on Site3

set S3-twoB shuffle S3-oneB

set S3-oneW sort [ who ] of wuggles-on Site3

set S3-twoW shuffle S3-oneW ]

end

to GP

ask buggles

[ ;;reproduce

repeat (random-poisson (mean-offspring))

[ hatch 1

[ set age (ticks + 1)

set homeland [ site-ID ] of patch-here

set mother [ who ] of one-of buggles-here with [age = ticks]

if (site-ID = 1)

[ let pos position mother S1-oneB

set father item pos S1-twoB ]

if (site-ID = 2)

[ let pos position mother S2-oneB

set father item pos S2-twoB ]

if (site-ID = 3)

[ let pos position mother S3-oneB

set father item pos S3-twoB ]

;;recombine

set recomb n-values 1 [ "M" ]

set recomb fput (bongo) recomb

repeat (network-size - 2)

[ set recomb fput (bongo2) recomb ]

set recomb reverse recomb

;;mutate

set heads map [ ifelse-value (random-float 1 > mutation-rate)

[ ? ]

[ random position ? heads ] ] heads

set fx map [ ifelse-value (random-float 1 > mutation-rate)

[ ? ]

[ random 2 ] ] fx

set chrom (sentence heads fx G)

;;calculate genotypes

set genotype n-values 1 [ G ]

repeat (network-size - 1)

[ set genotype fput "#" genotype

set genotype reverse genotype

set genotype replace-item (position "#" genotype) genotype (read2)

set genotype reverse genotype ]

set genotype reverse genotype

let max1 modes heads

let max1-div length max1

let max2 filter [ member? ? max1 ] heads

set od=max ((length max2 - 1) / max1-div)

;;calculate phenotype

set d=1 n-values network-size [ ? ]

foreach d=1 [ ifelse (member? (item ? tails) heads)

[ set d=1 replace-item ? d=1 0 ]

[ set d=1 replace-item ? d=1 1 ] ]

set phenotype (map [ ?1 * ?2 ] d=1 genotype )

set od=0 sum d=1

set P (sum phenotype * (140 / od=0 ))

;;calculate relative fitness

let delta (abs (P - ([ pH ] of patch-here)))

set rel-fit ( exp ((- 0.001) * (delta ^ 2)))

;;heritability

let MomP [P] of turtle mother

let PopP [P] of turtle father

set midP ((MomP + PopP) / 2) ] ] ]

ask wuggles

[ ;;reproduce

repeat (random-poisson (mean-offspring))

[ hatch 1

[ set age (ticks + 1)

set homeland [ site-ID ] of patch-here

set mother [ who ] of one-of wuggles-here with [age = ticks]

if (site-ID = 1)

[ let pos position mother S1-oneW

set father item pos S1-twoW ]

if (site-ID = 2)

[ let pos position mother S2-oneW

set father item pos S2-twoW ]

if (site-ID = 3)

[ let pos position mother S3-oneW

set father item pos S3-twoW ]

;;recombine

set recomb n-values 1 [ "M" ]

set recomb fput (bongo) recomb

repeat (network-size - 2)

[ set recomb fput (bongo2) recomb ]

set recomb reverse recomb

;;mutate

set heads map [ ifelse-value (random-float 1 > mutation-rate)

[ ? ]

[ random position ? heads ] ] heads

set fx map [ ifelse-value (random-float 1 > mutation-rate)

[ ? ]

[ random 2 ] ] fx

set chrom (sentence heads fx G)

;;calculate genotypes

set genotype n-values 1 [ G ]

repeat (network-size - 1)

[ set genotype fput "#" genotype

set genotype reverse genotype

set genotype replace-item (position "#" genotype) genotype (read2)

set genotype reverse genotype ]

set genotype reverse genotype

let max1 modes heads

let max1-div length max1

let max2 filter [ member? ? max1 ] heads

set od=max ((length max2 - 1) / max1-div)

;;calculate phenotype

set d=1 n-values network-size [ ? ]

foreach d=1 [ ifelse (member? (item ? tails) heads)

[ set d=1 replace-item ? d=1 0 ]

[ set d=1 replace-item ? d=1 1 ] ]

set phenotype (map [ ?1 * ?2 ] d=1 genotype )

set od=0 sum d=1

set P (sum phenotype * (140 / od=0 ))

;;calculate relative fitness

let delta (abs (P - ([ pH ] of patch-here)))

set rel-fit ( exp ((- 0.001) * (delta ^ 2)))

;;heritability

let MomP [P] of turtle mother

let PopP [P] of turtle father

set midP ((MomP + PopP) / 2) ] ] ]

end

to-report bongo

ifelse (random-float 1 < recombination-rate)

[ report "P" ]

[ report "M" ]

end

to-report bongo2

ifelse (item 0 recomb = "P")

[ ifelse (random-float 1 > recombination-rate)

[ report "P" ]

[ report "M" ] ]

[ ifelse (random-float 1 < recombination-rate)

[ report "P" ]

[ report "M" ] ]

end

to-report read2

let $h item (position "#" genotype) heads

let $f item (position "#" genotype) fx

let $s item $h genotype

ifelse (($s = 1 and $f = 1) or ($s = 0 and $f = 0))

[ report 1 ]

[ report 0 ]

end

to death

ask turtles

[ if age = ticks

[ die ]

if rel-fit <= random-float 1

[ die ] ]

if (count turtles-on Site1 > Site1-K)

[ ask turtles-on Site1

[ if (random (count turtles-on Site1) < (count turtles-on Site1 - Site1-K) )

[ die ] ] ]

if (count turtles-on Site2 > Site2-K)

[ ask turtles-on Site2

[ if (random (count turtles-on Site2) < (count turtles-on Site2 - Site2-K) )

[ die ] ] ]

if (count turtles-on Site3 > Site3-K)

[ ask turtles-on Site3

[ if (random (count turtles-on Site1) < (count turtles-on Site3 - Site3-K) )

[ die ] ] ]

end

to move

move-to one-of neighbors with [ pcolor != green ]

end

to disperse

ask buggles

[ if (random-float 1) < buggle-disp-pr

[ rt random 360

jump random 100 ]

if pcolor = green

[ die ]

ifelse (homeland = [site-ID] of patch-here)

[ set local "T" ]

[ set local "F" ] ]

ask wuggles

[ if (random-float 1) < wuggle-disp-pr

[ rt random 360

jump random 100 ]

if pcolor = green

[ die ]

ifelse (homeland = [site-ID] of patch-here)

[ set local "T" ]

[ set local "F" ] ]

end

to kill-program

if (count buggles < 2) or (count wuggles < 2) [ stop ]

end

to summary-info

;;Buggle global phenotype

set Bphen-list [P] of buggles

ifelse (length Bphen-list > 0)

[ set Bavg-phen mean Bphen-list ]

[ set Bavg-phen 0 ]

ifelse (length Bphen-list > 1)

[ set BVp variance Bphen-list ]

[ set BVp 0 ]

;;Buggle local phenotype

ifelse (count buggles-on Site1 > 1)

[ set bug_S1_P mean [P] of buggles-on Site1

set bug_S1_Vp variance [P] of buggles-on Site1 ]

[ set bug_S1_P 0

set bug_S1_Vp 0]

ifelse (count buggles-on Site2 > 1)

[ set bug_S2_P mean [P] of buggles-on Site2

set bug_S2_Vp variance [P] of buggles-on Site2 ]

[ set bug_S2_P 0

set bug_S2_Vp 0 ]

ifelse (count buggles-on Site3 > 1)

[ set bug_S3_P mean [P] of buggles-on Site3

set bug_S3_Vp variance [P] of buggles-on Site3 ]

[ set bug_S3_P 0

set bug_S3_Vp 0 ]

;;Wuggle global phenotype

set Wphen-list [P] of wuggles

ifelse (length Wphen-list > 0)

[ set Wavg-phen mean Wphen-list ]

[ set Wavg-phen 0 ]

ifelse (length Wphen-list > 1)

[ set WVp variance Wphen-list ]

[ set WVp 0 ]

;;Wuggle local phenotype

ifelse (count wuggles-on Site1 > 1)

[ set wug_S1_P mean [P] of wuggles-on Site1

set wug_S1_Vp variance [P] of wuggles-on Site1 ]

[ set wug_S1_P 0

set wug_S1_Vp 0]

ifelse (count wuggles-on Site2 > 1)

[ set wug_S2_P mean [P] of wuggles-on Site2

set wug_S2_Vp variance [P] of wuggles-on Site2 ]

[ set wug_S2_P 0

set wug_S2_Vp 0 ]

ifelse (count wuggles-on Site3 > 1)

[ set wug_S3_P mean [P] of wuggles-on Site3

set wug_S3_Vp variance [P] of wuggles-on Site3 ]

[ set wug_S3_P 0

set wug_S3_Vp 0 ]

;;Buggle heritability

set Bmp-list [midP] of buggles

if not empty? Bmp-list

[ set Bavg-MP mean Bmp-list ]

ask buggles

[ let BdevP (P - Bavg-phen)

let BdevMP (midP - Bavg-MP)

set num1 (BdevP * BdevMP)

set den1 (BdevMP * BdevMP) ]

let Bnum1-list [ num1 ] of buggles

set Bnumerator sum Bnum1-list

let Bden1-list [ den1 ] of buggles

set Bdenominator sum Bden1-list

if (Bdenominator > 0)

[ set BH2 (Bnumerator / Bdenominator) ]

;;Wuggle heritability

set Wmp-list [midP] of wuggles

if not empty? Wmp-list

[ set Wavg-MP mean Wmp-list ]

ask wuggles

[ let WdevP (P - Wavg-phen)

let WdevMP (midP - Wavg-MP)

set num1 (WdevP * WdevMP)

set den1 (WdevMP * WdevMP) ]

let Wnum1-list [ num1 ] of wuggles

set Wnumerator sum Wnum1-list

let Wden1-list [ den1 ] of wuggles

set Wdenominator sum Wden1-list

if (Wdenominator > 0)

[ set WH2 (Wnumerator / Wdenominator) ]

set BVg BH2 * BVp

set WVg WH2 * WVp

end
